# Multimodality palliative treatment with transarterial chemoembolization and high-intensity focused ultrasound for gastric leiomyosarcoma multiple liver metastasis pain: A case report

**DOI:** 10.1097/MD.0000000000017328

**Published:** 2019-09-27

**Authors:** Chien-shan Cheng, Lianyu Chen, Jing Xie, Zhen Chen

**Affiliations:** Department of Integrative Oncology, Fudan University Shanghai Cancer Center; Department of Oncology, Shanghai Medical College, Fudan University, Shanghai, China.

**Keywords:** case report, gastric leiomyosarcoma, high-intensity focused ultrasound, liver metastasis, transarterial chemoembolization

## Abstract

**Introduction:**

Gastric leiomyosarcoma (LMS) is a rare malignancy with minimal therapeutic options and has poor prognosis once metastasis develops.

**Patient concerns:**

A case of gastric LMS with multiple metastases, pain, and progressive anemia 13 months after the initial diagnosis in a 43-year-old woman.

**Diagnosis:**

Gastric LMS with liver metastases and multiple retroperitoneal lymphatic metastases.

**Interventions:**

Minimally invasive therapies of repeated tetrahydropalmatine and oxaliplatin-based transarterial chemoembolization and high-intensity focused ultrasound treatment were performed.

**Outcomes:**

The treatments resulted in significant pain relief (numerical rating scale from 8–2 points) after the initial treatment, improvement in performance status and quality of life, and a progression-free survival of 4 months after treatment.

**Conclusion:**

This combined modality palliative treatment approach was well tolerated with noticeable pain relief.

## 1 Introduction

Gastric leiomyosarcoma (LMS) accounts for about 1% of primary malignant tumors of the stomach, often metastasizes to the liver (60%), and has a poor prognosis.^[1–3]^ Almost all of LMS occur in adults, with the highest incidence in the sixth decade of life and the ratio of female to male is approximately 5:3.^[1,4]^ Unlike gastrointestinal stromal tumors, the only effective treatment for gastric LMS is surgical resection, and there is currently no effective molecular therapy.^[5]^ Although few case reports have suggested that repeated hepatectomy can prolong survival, appropriate therapeutic strategies remain controversial for patients with surgical resection contraindications or multiple hepatic and retroperitoneal lymph node metastases.^[6–8]^

Pain occurs in approximately 3-quarters of patients with advanced cancer, is one of the symptoms patients fear most, and greatly affects the quality of life in cancer survivorship.^[9–11]^ In patients with soft tissue sarcomas, pain is experienced in 53% of the patients and described as inadequately controlled in 63% of the patients based on a recent study.^[12]^ Increased evidence in oncology suggests that survival is associated with pain management.^[13]^ In palliative care, according to the World Health Organization (WHO), the means to provide prevention and relief of suffering of pain is an important measures.^[14]^ As a result, the further palliative therapeutic options of advanced LMS with contraindications for surgical resection is warranted, and the presentation of new cases is highly desirable.

## 2 Case presentation

**Figure 1:**
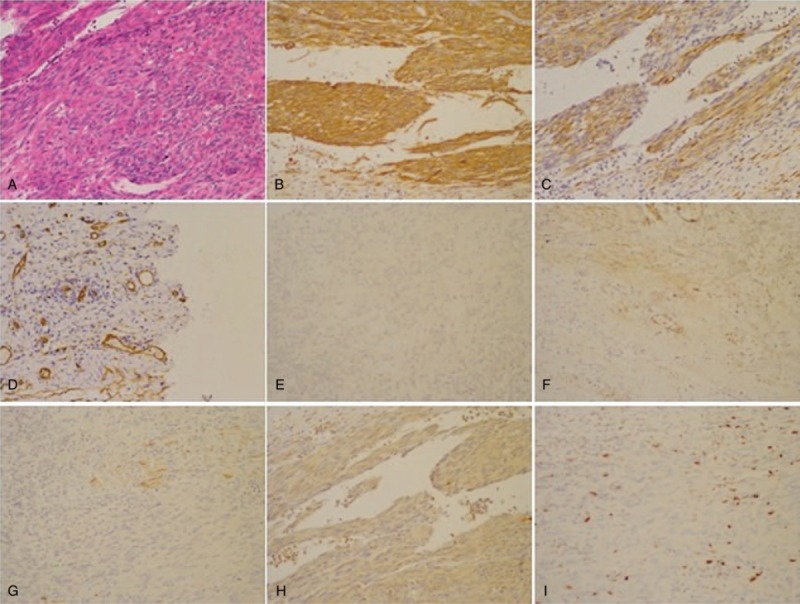
Immunohistological study of resected specimen. (A) Hematoxylin-eosin staining; (B) SMA (+); (C) calponin (partial+); (D) CD34 (−); (E) CD117 (−); (F) DOG (−); (G) desmin (±); (H) S-100 (−); and (I) Ki-67 (+) (About 15%). No KIT exon 9, 11, 13, 17, or PDGFRA exon 12 or 18 mutation. DOG = discovered on GIST-1, KIT = tyrosine-protein kinase KIT, PDGFRA = platelet—derived growth factor receptor alpha, SMA = smooth muscle actin.

**Figure 2:**
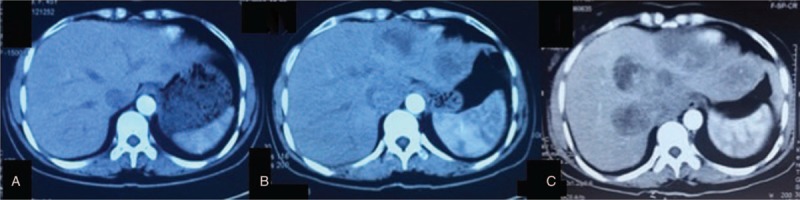
Enhanced CT scan (arterial phase) postsurgery at month 1, 3, and 11. (A) One month postoperatively. CT scan outlines a hypodense lesion suspicious of enlarged retroperitoneal lymph node. No treatment was performed. (B) Three months postoperatively. CT scan showed multiple new lesions in the left hepatic lobe with perilesion enhancement in arterial phase. No treatment was performed. (C) Eleven months postoperatively. CT scan showed progression of lesions in both hepatic lobes. Right hepatic lobe lesion progressed over 50 mm with obvious perilesion enhancement in arterial phase. No treatment was performed and patient was then referred to our hospital. CT = computed tomography.

A 43-year-old woman with unremarkable past medical history and no use of tobacco or alcohol presented with upper abdominal discomfort with extremity weakness. Upon admission to a local hospital, gastroscopy revealed a gastric mass of 3 cm in diameter. The Billroth I procedure was then performed 2 weeks later and the pathological diagnosis of the resected specimen was reported as a spindle cell tumor in the local hospital. Postoperatively, no adjuvant therapy was performed and the resected specimens were referred to our hospital for immunohistochemical examination. Histological and genetic examinations revealed a LMS (details shown in Fig. 1). Follow-up computed tomography (CT) scans are shown in Figure 2.

**Figure 3:**
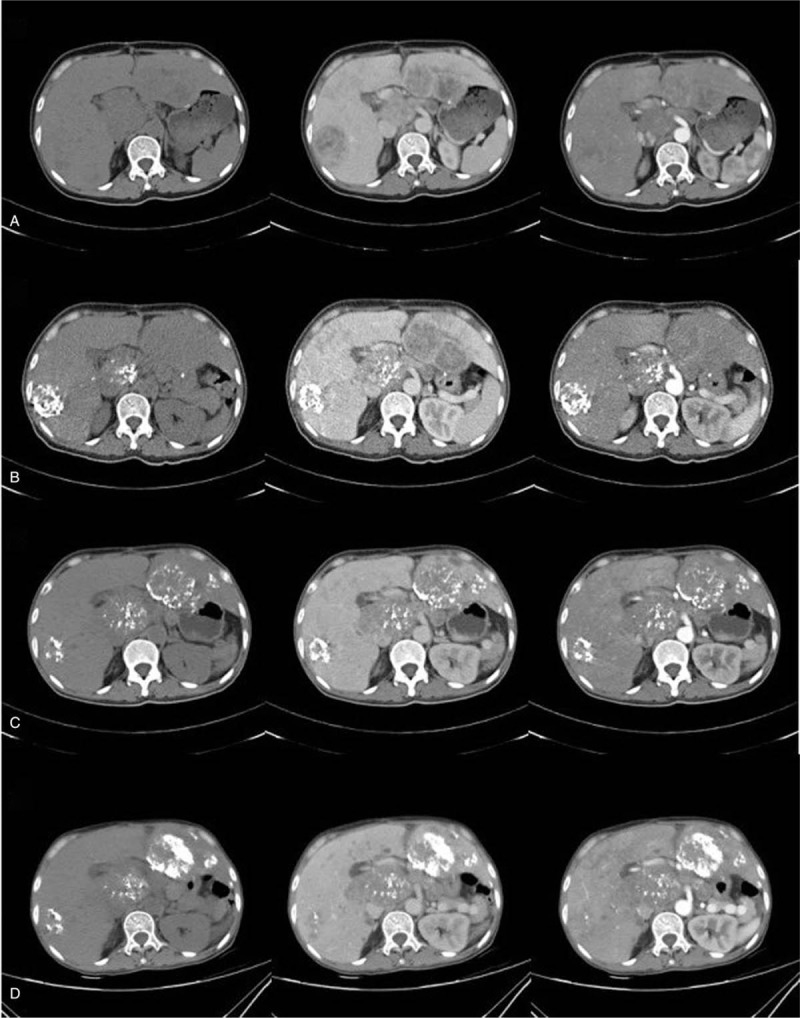
CT scan upon admission to our hospital. From left to right are plain CT scans, venous phase and arterial phase, respectively. (A) First admission, multiple lesions (maximal diameter of 55 mm) with perilesion venous and arterial enhancement. (B) Second admission, 2-month post TACE treatment. Lipiodol deposition was visible. Lesion on segment V and VIII regressed to 35 mm in diameter. Clinically assessed as SD. (C) Third admission, 2-month post 2nd TACE treatment. Lesion in the right hepatic lobe further regress and lipiodol deposition was visible. Clinically assessed as SD. (D) Three-month post 3rd TACE treatment. Multiple newly developed lesions in both left and right hepatic lobe, vena cava, and portal vein compression by hepatic lesions, ascites, and bilateral pleural effusion. Clinically assessed as disease progression. CT = computed tomography, SD = stable disease, TACE = transarterial chemoembolization.

The patient was referred to our hospital 1 year after surgery, complaining of upper abdominal discomfort and low back pain (numerical rating scale, NRS of 8). Upon admission, the patient presented with severe anemia with a hemoglobin (Hb) of 65 g/L and a Karnofsky status (KPS) 70, swelling, loss of appetite, and significant weight loss (58–43 kg, 160 cm, body mass index 22.65–16.79). Plain CT scan showed multiple scattered hypodense hepatic lesions with a maximum diameter of 5.5 cm, nonhomogeneous enhancement, suspicious retroperitoneal lymphadenopathy, and multiple portal vein lymphadenopathy. Based on these findings, the patient was diagnosed with a gastric LMS with multiple liver metastases and lymph node metastases (T3N0M0→rM1, stage IV), as shown in Figure 3A.

**Figure 4:**
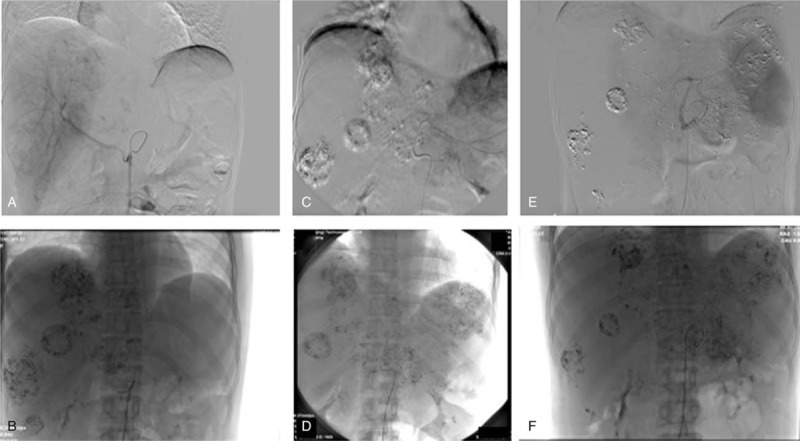
DSA and TACE treatment. (A) DSA in 1st TACE treatment. Chemoinfusion and arterial embolization of L-OHP 150mg and THP 50 mg mixed with 10 mL lipiodol (Lp) was performed only to right hepatic mass through superior mesenteric artery and heterotopic hepatic artery. (B) Lipiodol deposition was visible intraprocedurally. (C) DSA in 2nd TACE treatment. Via left hepatic artery, same regimen (L-OHP 150 mg + THP 50 mg + Lp 10 mL) was injected. (D) Lipiodol deposition was visible intraprocedurally. (E) DSA in 3rd TACE treatment. Via proper hepatic artery, same regimen (L-OHP 100 mg + THP 30 mg + Lp 15 mL) was injected. (F) Lipiodol deposition was visible intraprocedurally. DSA=digital subtraction angiography, L-OHP = oxaliplatin, TACE = transarterial chemoembolization, THP = tetrahydropalmatine.

**Figure 5:**
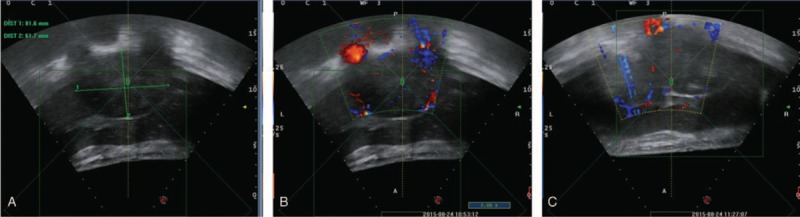
HIFU treatment. (A) Pretreatment ultrasound of the retroperitoneal lymph node lesion showed hypoechogenicity. (B) Pretreatment ultrasound of the retroperitoneal lymph node lesion with illustration of blood supply. (C) Post-HIFU treatment of the lesion showed hyperechogenicity. HIFU = high-intensity focused ultrasound.

Considering that the patient's physical performance and general condition may not be able to tolerate systemic chemotherapy or hepatectomy, we performed Seldinger technique transarterial chemoembolization (TACE) with oxaliplatin (L-OHP) and tetrahydropalmatine (THP) emulsion with lipiodol.^[15]^ Intra-arterial digital subtraction angiography showed a diffuse peritumoral vascularization of lesions located in both left and right hepatic lobes, as shown in Figure 4. The chemoinfusion and arterial embolization regimen are described in detail in Figure 4. Subsequently, high-intensity focused ultrasound (HIFU) treatment was performed on the retroperitoneal lymphatic metastases. The size of the lesion undergone HIFU treatment was 80 × 70 × 60 mm; HIFU treatment frequency was 1 MHZ, with a focal length of 151 mm. A total of 12 layers with distance of 5 mm were treated with a sum treatment time of 729 seconds. The average power of HIFU treatment was 215 W and the total treatment energy 157,040 J. After HIFU treatment, the center position of the lesion is obvious before and after treatment, as shown in Figure 5. A total of 3 times of TACE were performed within 5 months and were clinically assessed as stable disease according the response evaluation criteria in solid tumors (RECIST).^[16]^ The patient tolerated the procedures well and no obvious complications (greater than grade 1, Common Terminology Criteria for Adverse Events v5.0) were observed. The follow-up CT scans showed visible lipiodol deposition and local tumor control (details and RECIST response are described and shown in Fig. 3B and C).

During treatment, the patient complained of significant pain relief after initial treatment (NRS from 8–2 points). KPS improved from 70 to 80. Also, the symptoms of swelling and nausea reduced, appetite improved, and body weight increased (from 43–48 kg). Three months later, upon follow-ups, the patient developed loss of appetite, malnutrition, extremities weakness, and severe anemia (Hb 45 g/L). The patient complained of xiphoideusal pain while abdominal CT scan showed disease progression with multiple new lesions in both left and right hepatic lobes, as shown in Figure 3D. Blood transfusion and palliative care were provided for symptom relief. The patient survived for another 2 months and survived a total of 20 months after the first diagnosis of gastric lesion due to tumor progression.

## 3 Discussion

### 3.1 Treatment

Surgery is the main treatment for gastric LMS, and the survival rate is closely related to the type of surgery, the extent, and size of tumor lesions. Extensive surgical resection and regional lymph node dissection are considered as the preferred treatment of choice for gastric LMS.^[6,7]^ For smaller tumors, less than 2 cm in diameter, a wide local excision and a surgical edge of more than 3 cm from the tumor edge is preferred.^[17,18]^ For larger tumors, more than 2 cm in diameter, gastrectomy and total gastrectomy combined with gastric lymph node resection are the 2 main surgical approaches.^[17,18]^ It has been suggested in the literature that D1 gastrectomy with lymphadenectomy has a lower recurrence rate and a better prognosis than simple gastrectomy.^[19]^

The liver and the abdominal cavity are the 2 most common sites of recurrence and metastasis of LMS. Even though surgical resection of the metastatic lesions is considered as the primary treatment and can significantly prolong overall survival to a 30% of 5-year survival after resection of the metastatic disease^[2]^; surgical contradictions such as inadequate liver function, metastatic tumor location, size, and number of lesions limit surgical intervention and/or lead to a higher recurrence rate after resection. Although some literature suggests that repeated hepatectomy for frequently recurrent liver metastases may prolong survival, most patients often lose the opportunity for radical surgery once the tumor recurs.^[6–8]^ In this patient, severe anemia and low body weight are contraindications for surgical resection; also, hepatic metastases are accompanied by lymphatic metastases. The treatment approach for this group of patients deserves further discussion.

### 3.2 Role of TACE in recurrent LMS with hepatic metastases

TACE is widely used to treat liver tumors, especially for unresectable liver tumors.^[20,21]^ Compared with systemic chemotherapy, TACE has the advantage of increasing the drug delivery efficacy, yielding a higher concentration of drug in tumors, reducing systemic side effects, killing tumor cells selectively, embolizing vascular supply to inhibits tumor growth, and allowing repeated treatment of liver lesions.^[22,23]^ LMS is generally believed as a hypervascular tumor with abundant blood supply from the hepatic artery and the absence of hepatic portal vein tumor thrombus, and therefore enables complete embolization by TACE and improves overall therapeutic outcomes.^[24]^

Currently, the treatment outcome of TACE in LMS lacks large-scaled clinical observations. Mavligit et al^[25]^ reported 14 cases of TACE-treated LMS liver metastases and suggested a significantly superior treatment outcome than systemic chemotherapy. As there are no specific tumor biomarkers for LMS, imaging investigation have become particularly crucial in both monitoring the disease and assessing the treatment outcomes. The treatment outcomes of TACE are closely related to the degree of vascularity in tumors. We suggest that enhanced CT scan and hepatic angiography to observe changes in blood supply before and after embolization, as well as to determine the presence of new lesions have significant clinical value for patients with LMS liver metastases.

On the other hand, controversies remain about whether embolization alone or in combination with chemotherapy yields better treatment outcomes. It has been reported simple hepatic arterial infusion chemotherapy is not effective in the treatment of LMS liver metastasis and sole local embolization of sarcoma metastases can significantly improve the survival of patients.^[26,27]^ Although it has been suggested that LMS is not sensitive to chemotherapeutic drugs, it may only increase adverse reactions.^[26]^ Our experience in combining L-OHP and THP emulsion with lipiodol has received satisfactory outcomes with minimal hepatotoxicity in gastrointestinal tumor with liver metastasis. For patients with unresectable tumor, transarterial embolization either with or without chemotherapy drugs can effectively control the progress of LMS, improve the clinical symptoms by reducing tumor blood supply, and prolong the survival time. As a minimally invasive treatment, TACE provides treatment opportunities for patients with surgical contraindications, such as severe anemia, and improves the patient's quality of life (such as pain) as a palliative treatment for advanced tumors.

### 3.3 HIFU treatment and its role in metastatic lesions

HIFU is a novel and completely noninvasive local ablation method that uses focused ultrasound energy from an extracorporeal source to a target within the body and destroys tumor cells by tissue ablation without the need for a surgical incisions.^[27]^ HIFU has been successfully used in preclinical trials for palliative care in patients with the aim of improving quality of life, controlling pain, improving performance status, and prolonging survival.^[27]^ HIFU is a relatively safe procedure because it is noninvasive and can be applied in the patient's full consciousness. There are no serious complications or adverse events associated with HIFU treatment.^[27]^ From a technical point of view, noninvasive ablations of deep tissue targets using HIFU treatment is feasible and safe.

### 3.4 Roles of palliative treatments in LMS survival and pain management

At present, there is a lack of standardized chemotherapy for LMS. In advanced soft tissue sarcoma, the median survival of systemic palliative first-line chemotherapy, mostly with doxorubicin, in patients treated on routine palliative protocols ranges between 7 and 12 months.^[28–30]^ Further, the objective response rate to combined chemotherapy, mostly doxorubicin and an anthracycline combined with an alkylator, was only 20% and the median progression-free survival (PFS) was 4.9 months (range 0.1–17.1).^[31]^ Moreover, 50% of patients starting first-line chemotherapy experienced pain; and among them, 20% of patients reported suffering from uncontrolled pain despite following the WHO 3-step analgesic ladder.^[32]^ In the present case, although the patient did not present with comorbidities such as cardiopulmonary and renal dysfunctions, the severe anemia is associated with fatigue, loss of appetite, and decrease in quality of life. Best supportive care was provided in addition to the multimodality palliative treatment with TACE and HIFU treatment resulting in a PFS of 4 months and overall survival of 20 months.

According to our experience, repeated TACE intervention achieved good therapeutic effects with minimal adverse effects on metastatic liver tumors. In this case, the patient experienced a decrease in NRS pain scores from 8 to 2 without affecting liver function or inducing apparent side effects. Vital organs and liver function were well protected during the multimodality treatment. We concluded that the multimodality palliative treatment approach with repeated TACE and HIFU treatments can be well tolerated by patients with severe anemia, who are generally in a poor physical condition and improve their quality of life.

## Acknowledgments

The authors would like to thank Dr Jie Chen, Dr Yehua Shen, Dr Kun Wang, and Dr Jingxian Chen for their advice in the preparation of this manuscript.

## Author contributions

**Conceptualization:** Chien-shan Cheng, Zhen Chen.

**Formal analysis:** Lianyu Chen, Jing Xie.

**Investigation:** Jing Xie.

**Supervision:** Zhen Chen.

**Validation:** Lianyu Chen.

**Writing – original draft:** Chien-shan Cheng.

**Writing – review & editing:** Chien-shan Cheng, Lianyu Chen, Jing Xie, Zhen Chen.

Chien-shan Cheng orcid: 0000-0003-4885-6759.

Lianyu Chen orcid: 0000-0003-3731-8592.

Jing Xie orcid: 0000-0003-2566-7250.

Zhen Chen orcid: 0000-0002-4502-0801.
